# Creating a Live and Flexible Normative Dataset for Netball

**DOI:** 10.3389/fspor.2021.743612

**Published:** 2021-10-22

**Authors:** Hayden Croft, Kirsten Spencer, Noeline Taurua, Emily Wilton

**Affiliations:** ^1^Institute of Sport Exercise and Health, Otago Polytechnic, Dunedin, New Zealand; ^2^Sports Performance Research Institute New Zealand, Auckland University of Technology, Auckland, New Zealand; ^3^Netball New Zealand, Auckland, New Zealand

**Keywords:** netball, performance analysis, coaching (performance), normative data, big data, analytics

## Abstract

A previous research has identified large data and information sources which exist about netball performance and align with the discussion of coaches during the games. Normative data provides context to measures across many disciplines, such as fitness testing, physical conditioning, and body composition. These data are normally presented in the tables as representations of the population categorized for benchmarking. Normative data does not exist for benchmarking or contextualization in netball, yet the coaches and players use performance statistics. A systems design methodology was adopted for this study where a process for automating the organization, normalization, and contextualization of netball performance data was developed. To maintain good ecological validity, a case study utilized expert coach feedback on the understandability and usability of the visual representations of netball performance population data. This paper provides coaches with benchmarks for assessing the performances of players, across competition levels against the player positions for performance indicators. It also provides insights to a performance analyst around how to present these benchmarks in an automated “real-time” reporting tool.

## Introduction

A previous research (Croft et al., 2020) has identified tactical themes that netball coaches discuss during the matches and subsequently data from sports performance analysis tools that align with these themes have been evaluated. Public domain data providers, such as Champion Data™ (Victoria, Australia) or notational analysis software, such as Hudl-Sportscode (Lincoln, USA), allow access to this data *via* either application programming interfaces (APIs), (in full) or live coding of events, respectively. The twin benefits of this are that the data are generated in real-time and immediately presented to the coach courtside, enabling the coach to reflect-in-action enhancing the professional practice and learning (Edwards, 2017). Therefore, it is essential that the data are presented in a way that is understandable and have strong face validity to assist coach decision-making during a match. The historical data and information may provide context to this live data, specifically by indicating whether the current-performance is above or below the relative past-performance, i.e., in comparison with the normative data.

### Normative Data

The importance of normative data (O'Connor, 1990) to aid the establishment of standards of excellence for physicians is standard procedure in medical research. The sport physiology research has developed and utilized normative data, predominately for movement screening (Fox et al., 2014), fitness assessment (Tomkinson et al., 2018), anthropometry (body mass index [BMI], skinfolds), and physical conditioning (submaximal VO2 max).

A performance analysis research (O'Donoghue, 2005) has developed normative profiles, although these studies have presented normative data tables that generally are fixed and only represent the performances from a set period for a specific population.

There are currently no standardized normative datasets present in the netball literature. A recent research (Bruce et al., 2018) has identified the performance indicators that differ between the competitions, or those that are related to winning. However, this research does not provide coaches with usable measures that give context to the performance of an individual. Thus, the development of normative data tables for netball would enable the player or the coach to understand if their current performance, for example, goal volume, was relatively high, moderate, or low. This fixed normative dataset could also be used as a tool in the player development pathways to assist with identifying areas of strength and/or weakness in a player, and for the rehabilitation targets if recovering from an injury. However, the comparisons with a fixed dataset may need to be viewed with caution, as the performance trends and strategies are dynamic constructs. The solution is to create a dynamic normative data table from an automated database that continually updates as performances are added.

### Databases and Data Management

**Figure 1:**
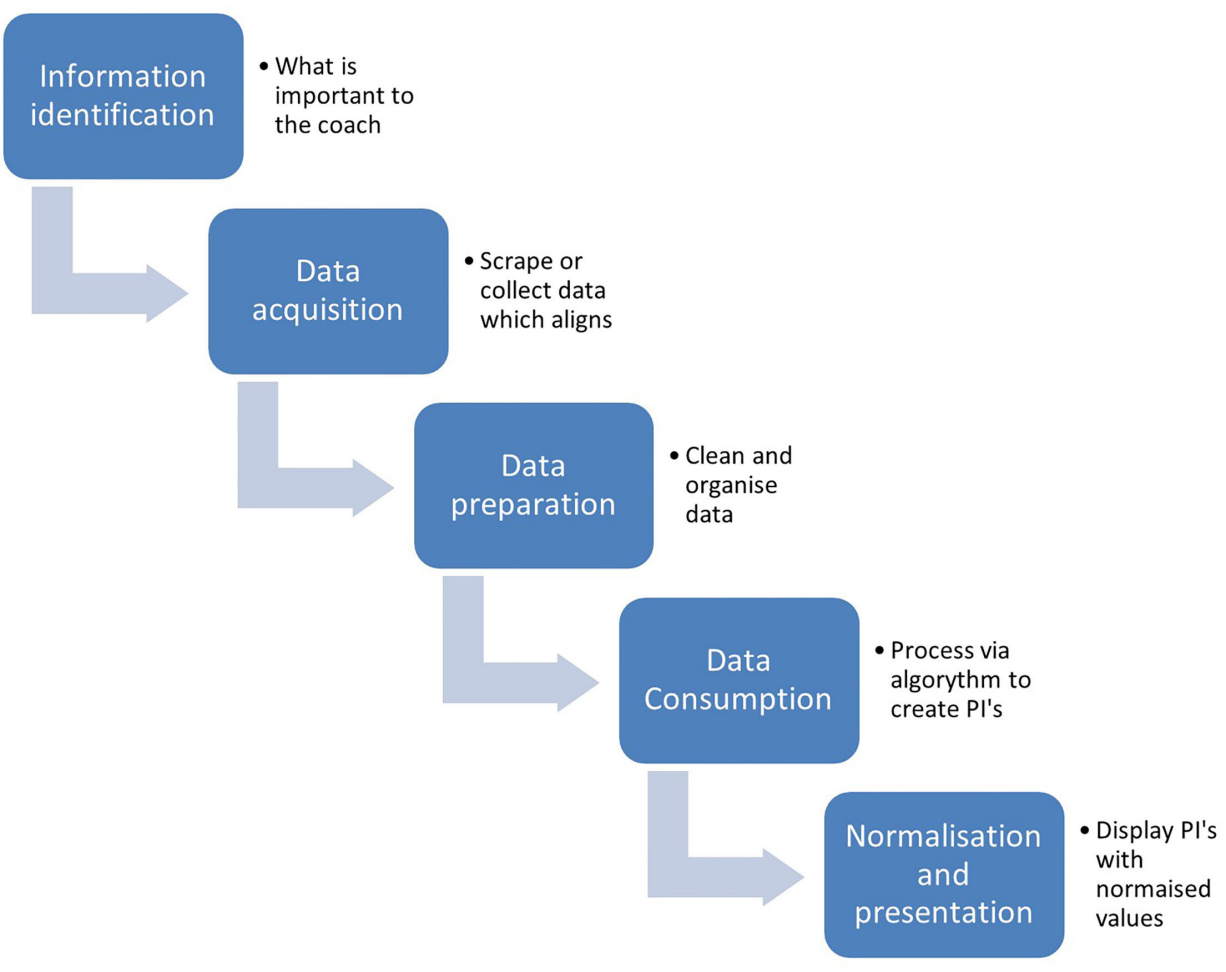
Pipeline for automated data management for display in visualisation tools.

A database combined with other data processing tools (Figure 1) appears to be the most obvious solution with some literature having focused on their design specifically for sport science data. Vincent et al. (2009) overviewed the design and implementation of databases in sport with specific focus given to their use as a performance analysis tool. They summarized the most effective use of databases, data management, and content management systems as an answer to the subjective reliance of the coaches on “gut feel” or intuitive thinking about performance. The complex nature of navigating databases and creating simple interfaces for coaches that allows them to easily interpret, coordinate, and implement the information within should be considered.

In their simplest form, a database can be a spreadsheet much like those built in Microsoft Excel™ (Redmond, USA). In contrast, they can also be very complex with the products available that allow capturing, storage, retrieval, interpretation, reporting, and dissemination of information, for example, Fusion-Sports_inc_ (Queensland, Australia) Smart abase™ athlete management system. A good database design is crucial for usability, however, if the data entered is not accurate or valid then they can produce false information. Two possible data entry methods (Vincent et al., 2009) are:

1. An automated computer algorithm that enables fast inputting as data are created without the checks for accuracy.

2. Manual inputting that can be slower but more accurate.

This statement is somewhat generalized as it could be argued that a well-designed, automated process, with validity, reliability, and accuracy checks (O'Donoghue, 2015) could be more accurate than manual inputting which is susceptible to human error. As this study will look to automate as much of the process possible, the checks for accuracy need to be integrated *via* validation conditions. The well-designed databases or data management systems should avoid unnecessary redundancy and duplication of information. These recommendations are based on a relational model for databases (Codd, 1970) that involves organizing data into tables of rows and columns.

### Sources of Data and Variable Selection

The themes identified by Croft et al. (2020) were utilized in this study and focused on the movement, positioning, actions, possession outcomes, and strategy, with the layers of lower and higher order themes. Publicly available information (Champion Data™) and coded information produced using a notational analysis software [HudlSportscode (Lincoln, USA)™, Dartfish (Fribourg, France)™, and Nacsport (Las Palmas de Gran Canaria, Las Palmas)™] that were best suited for use in a dynamic database tool. This was due to them having good face and ecological validity could be accessed and organized easily and as publicly available information, such as Champion Data (Victoria, Australia)™ have several years of historical data available, information can be scraped manually or sourced *via* an Extensible Mark-up Language (XML) file. This research has aligned existing data sources to the tactical themes that coaches discuss during the games. By incorporating expert coach feedback into the design of data visualizations or data tables ecological validity should be maintained across the design process.

The purpose of this study is to design a platform for capturing, processing, and organizing data so it can be presented as a normative data table. These normative data tables will provide context for players about their performance against the historical performances at the same level of competition and position played. Expert coach feedback will be used to guide how these data are best visualized and to maintain ecological validity.

## Materials and Methods

### Equipment

A personal computer device connected to high-speed internet was used to upload the XMLs and comma-separated values (CSV) file type to an AWS S3–Amazon™ (Seattle, United States) Simple Storage Service (a cloud storage service). Event-driven pipelines within the AWS Amazon Website Service suite, processed the data, integrating and transforming it and making it ready for consumption in an analysis software tool Tableau™ (Seattle, United States). Tableau™ (Seattle, United States) was connected to this database and a normative data table was built to summarize means, SDs, and percentiles for each level of competition and position.

### Procedures

#### Data Source

The data were sourced from the coded XML files produced in Hudl-Sportscode™ (Lincoln, USA) and a public domain website (Champion Data, 2020). The data were collected between 2014 and 2020 from: International netball matches, ANZ Premiership™ (formally Championship), Suncorp Super Netball™, Beko National Netball League™, New Zealand (NZ) U23 matches, NZ U19 matches, and NZ Secondary Schools competitions. ~20,320 rows of individual player data were collated.

#### Data Acquisition

Both manually scraped and coded XML data sources were collected and loaded into the relational database. The database accepted this data in both the raw XML format, which required processing *via* an algorithm to extract the variables in a format useable within the database. The database was also designed to accept the scraped data within a CSV file. This data was organized into a single-paged matrix format, with one performance per player, per row and variables organized into columns. These samples were tested for completeness and input errors.

#### Data Preparation and Cleaning

As scraped and coded data required large repetition of inputting as well as dimensions being added manually, such as competition, team, and year, data cleaning was required to ensure an accurate dataset. This was achieved in two ways, firstly, visually observing each row of data for missing, misaligned, and obviously incorrect numbers. Secondly, the data were graphed and checked for averages >0 in the categories where a player could not have achieved a frequency, i.e., a center cannot attempt a goal. The data that were identified as incorrect were either corrected or removed from the dataset. Most errors appeared to be incorrect due to the wrong input of the player position.

#### Upload and Consumption

Once the dataset was complete, the XMLs and.csv tables were imported into the AWS S3–Amazon (Seattle, United States) Simple Storage Service cloud storage and an event-driven pipeline automatically transformed the data ready to be consumed by the Tableau™ dashboard. This process of transformation and consumption takes a few seconds depending on the internet connections and dataset size. A Tableau™ (Seattle, United States) dashboard was designed to allow the dataset to be filtered by competition level, such as international, national (ANZ Premiership and Beko), Australian national, NZ under 23 years, NZ under 19 years, and secondary school notational tournament. A filter was also built to allow the selection of the year of the performance and the teams competing. This allows the user the ability to select more specific populations, if required.

#### Normalization and Presentation

Once in the Tableau™ (Seattle, United States) dashboard, further transformation of the data was completed. The performance measures were normalized into per 60 min played. This was done so that all the performances could be compared across competitions, regardless of the time on court. In this, 60 min is chosen as this is the normal length of an international netball match. For presentation on the normative table, the percentiles and medians were used (Hopkins, 1997). When the data is not of a normal distribution, i.e., there are a lot of performances at the larger end of the scale, an average would not accurately represent the dataset. For this study, 25th, 50th, and 75th percentiles were chosen, however, 90th and 95th percentiles are easily generated using the features of the software.

## Results

**Figure 2:**
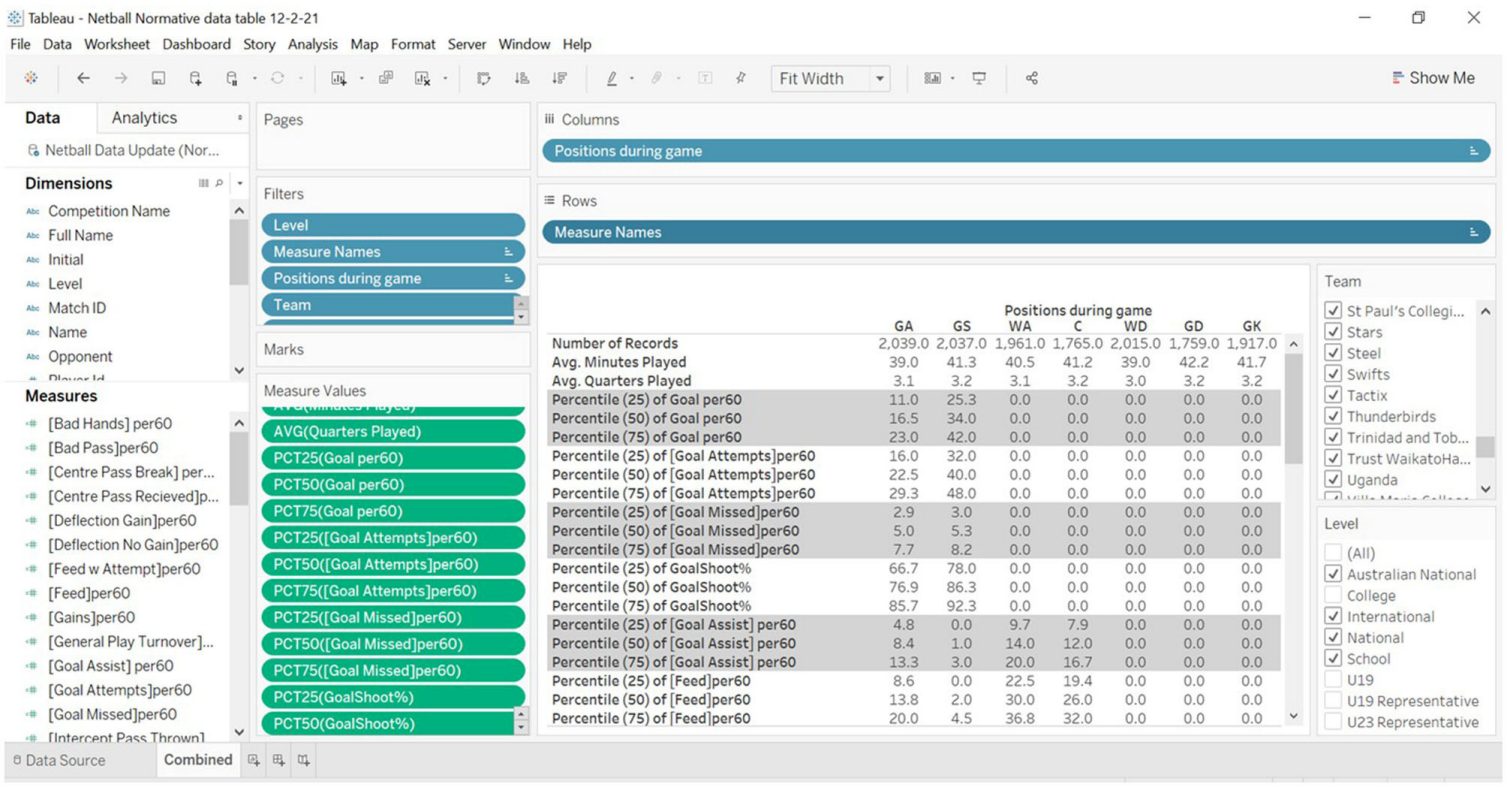
Layout of Tableau™ reporting interface with filters (right side) for competition, level, and year present.

The normative data table (Figure 2) presented here is divided into vertical layers, or clustered rows, for each level of competition, and then into columns for each playing position. This allows easy navigation by the user and comparisons for each position. The positions that are similar in their roles, i.e., Goal Shot and Goal Attack, are ordered across the columns for ease when compared.

The number of samples (*n* = 1) in each competition level is also listed to allow the user to understand the size of the population they are comparing against. This population size automatically varies dependent on the additional matches being uploaded into the cloud database or the filtering option that is selected, i.e., years, months, and days. The normative data table presented below displays all the data, excluding those with the missing data that have been loaded into the database. The time scale for these matches occurred between the dates of 01 January 2014 and 01 July 2020.

The Dimensions under the Data tab (far top left) can be used to create filters which appear on the left and in the boxes on the far right. These dimensions include Competition Name, Level, Measure Names, Positions During Game, Team, and Year. However, for this table only Team, Position During Game and Level have been used. In the list of Measures on the left side, a new calculated variable was created that represented the given measure as a value scaled to represent 60 min of play. This normalized all values to enable direct comparison, and they were then presented in the table as 25th, 50th (median), and 75th percentiles or interquartile ranges (Hopkins, 1997). These can be easily adjusted to represent other percentiles, i.e., 5th, 10th, 90th, and 95th.

For reporting in this paper, this table was exported into Microsoft Excel™ (Microsoft Corporation, WA, USA) and is presented below in Table 1. Table 1 shows, after cleaning and filtering that there were 2,039 records, or player performances, for Goal Attacks (GA), 2,037 records for Goal Shoot (GS), 1,961 for Wing Attack (WA), 1,765 for Centre (C), 2,015 for Wing Defence (WD), 1,759 for Goal Defence (GD), and 1,917 for Goal Keep (GK). The performances where a player shifted between two or more positions during a game were excluded.

**Table 1:** Combined normative data of international, national, and school level netball performance indicators.

**Position**	**GA**	**GS**	**WA**	**C**	**WD**	**GD**	**GK**
Number of Records	2,039.0	2,037.0	1,961.0	1,765.0	2,015.0	1,759.0	1,917.0
Avg. minutes played	39.0	41.3	40.5	41.2	39.0	42.2	41.7
Avg. quarters played	3.1	3.2	3.1	3.2	3.0	3.2	3.2
Percentile (25)	11.0	25.3	0.0	0.0	0.0	0.0	0.0
Percentile (50) of [goals] per 60	16.5	34.0	0.0	0.0	0.0	0.0	0.0
Percentile (75)	23.0	42.0	0.0	0.0	0.0	0.0	0.0
Percentile (25)	16.0	32.0	0.0	0.0	0.0	0.0	0.0
Percentile (50) of [goal attempts]	22.5	40.0	0.0	0.0	0.0	0.0	0.0
Percentile (75)	29.3	48.0	0.0	0.0	0.0	0.0	0.0
Percentile (25)	2.9	3.0	0.0	0.0	0.0	0.0	0.0
Percentile (50) of [goal missed] per 60	5.0	5.3	0.0	0.0	0.0	0.0	0.0
Percentile (75)	7.7	8.2	0.0	0.0	0.0	0.0	0.0
Percentile (25)	66.7	78.0	0.0	0.0	0.0	0.0	0.0
Percentile (50) of goal shoot %	76.9	86.3	0.0	0.0	0.0	0.0	0.0
Percentile (75)	85.7	92.3	0.0	0.0	0.0	0.0	0.0
Percentile (25)	4.8	0.0	9.7	7.9	0.0	0.0	0.0
Percentile (50) of [goal assist] per 60	8.4	1.0	14.0	12.0	0.0	0.0	0.0
Percentile (75)	13.3	3.0	20.0	16.7	0.0	0.0	0.0
Percentile (25)	8.6	0.0	22.5	19.4	0.0	0.0	0.0
Percentile (50) of [feed] per 60	13.8	2.0	30.0	26.0	0.0	0.0	0.0
Percentile (75)	20.0	4.5	36.8	32.0	0.0	0.0	0.0
Percentile (25)	7.5	0.0	15.0	13.0	0.0	0.0	0.0
Percentile (50) of [feed w attempt] per 60	12.0	1.4	21.0	18.0	0.0	0.0	0.0
Percentile (75)	18.0	3.9	27.0	22.2	0.0	0.0	0.0
Percentile (25)	12.0	0.0	14.0	0.0	2.0	2.0	0.0
Percentile (50) of [centre pass received] per 60	16.7	0.0	19.5	0.0	4.2	4.0	0.0
Percentile (75)	21.7	0.0	24.9	0.0	7.5	7.2	0.0
Percentile (25)	0.0	0.0	0.0	0.0	0.0	1.9	2.9
Percentile (50) of [gains] per 60	0.0	0.0	0.0	1.0	1.3	3.5	5.0
Percentile (75)	1.0	0.0	1.0	2.0	2.9	5.9	7.5
Percentile (25)	0.0	0.0	0.0	0.0	0.0	0.0	0.0
Percentile (50) of [intercept] per 60	0.0	0.0	0.0	0.0	1.0	1.5	2.0
Percentile (75)	0.0	0.0	0.0	1.5	2.0	3.0	3.9
Percentile (25)	0.0	0.0	0.0	0.0	0.0	0.0	0.0
Percentile (50) of [deflection gain] per 60	0.0	0.0	0.0	0.0	0.0	1.5	1.9
Percentile (75)	1.0	0.0	0.0	1.8	2.0	3.9	4.0
Percentile (25)	0.0	0.0	0.0	0.0	0.0	1.0	1.4
Percentile (50) of [deflection no gain] per 60	0.0	0.0	0.0	1.0	1.5	2.9	3.0
Percentile (75)	1.0	0.0	1.0	2.0	3.0	4.0	5.0
Percentile (25)	0.0	0.0	0.0	0.0	0.0	0.0	0.0
Percentile (50) of [pick up] per 60	1.0	0.0	1.1	1.7	1.1	1.1	1.0
Percentile (75)	2.0	1.4	2.6	3.0	2.9	2.6	2.0
Percentile (25)	0.0	0.0	0.0	0.0	0.0	0.0	0.0
Percentile (50) of [rebound] per 60	0.0	2.0	0.0	0.0	0.0	1.0	1.5
Percentile (75)	1.5	3.9	0.0	0.0	0.0	2.0	3.0
Percentile (25)	0.0	0.0	1.0	3.6	4.5	6.0	7.0
Percentile (50) of [penalty contact] per60	2.1	1.7	2.9	6.0	7.3	9.0	10.0
Percentile (75)	4.4	3.0	5.0	9.0	10.9	12.0	14.5
Percentile (25)	0.0	0.0	0.0	0.0	0.0	1.5	2.0
Percentile (50) of [penalty obstruction] per 60	0.0	0.0	0.0	1.1	1.5	3.0	4.0
Percentile (75)	1.3	1.0	1.2	3.0	3.0	5.9	6.7
Percentile (25)	3.0	3.0	2.0	1.5	0.0	0.0	0.0
Percentile (50) of [general play turnover] per 60	5.0	4.5	4.0	3.0	1.0	1.0	0.0
Percentile (75)	7.5	7.0	6.0	5.2	2.7	2.3	1.0
Percentile (25)	0.0	0.0	0.0	0.0	0.0	0.0	0.0
Percentile (50) of [bad pass] per 60	0.0	0.0	0.0	0.0	0.0	0.0	0.0
Percentile (75)	1.5	0.0	1.9	1.5	0.0	0.0	0.0
Percentile (25)	0.0	0.0	0.0	0.0	0.0	0.0	0.0
Percentile (50) of [intercept pass thrown] per 60	1.0	0.0	1.3	1.2	0.0	0.0	0.0
Percentile (75)	2.0	0.0	2.9	2.6	1.1	1.0	0.0
Percentile (25)	0.0	0.0	0.0	0.0	0.0	0.0	0.0
Percentile (50) of [centre pass break] per 60	0.0	0.0	0.0	0.0	0.0	0.0	0.0
Percentile (75)	0.0	0.0	0.0	0.0	0.0	0.0	0.0
Percentile (25)	0.0	0.0	0.0	0.0	0.0	0.0	0.0
Percentile (50) of [bad hands] per 60	0.0	1.0	0.0	0.0	0.0	0.0	0.0
Percentile (75)	1.3	2.0	1.1	0.0	0.0	0.0	0.0
Percentile (25)	0.0	0.0	0.0	0.0	0.0	0.0	0.0
Percentile (50) of [offside] per 60	0.0	0.0	0.0	0.0	0.0	0.0	0.0
Percentile (75)	0.0	0.0	0.0	1.0	1.0	0.0	0.0

Some of the results presented in Table 1 include, GS's taking more shots at goal (34 vs. 16.5 per 60 min) than GA's and taking these at a higher goal shooting percentage (86.3 vs. 76.9%). WA's were the most frequent with feeds (30 vs. 26 per 60 min) and Goal Assist (14 vs. 12 per 60 min). They also had higher Centre Pass Receives than any other position that include GA's (19.5 vs. 16.7 per 60 min). As the GA has a role of receiving centre passes, feeding the circle, and shooting goals, it is logical that they have lower values for these than the more specialized positions of WA and GS. The C position also has a broader role in both attack and defence and therefore has lower values than the WA for feeds (26 vs. 30 per 60 min) and Goal Assists (12 vs. 14 per 60 min). In comparison with the defensive midcourt position WD for gains (1.0 vs. 1.3 per 60 min). The C position does however have the highest frequency of pick up (1.7 vs. 1.1 per 60 min) which could be due to them being allowed to cover more area of the court than any other position.

In the defensive area of the court, we see a similar relationship between the GK and the GD as to that of the GS and GA in the attacking end. For defensive measures, Intercepts (2.0 vs. 1.5 per 60 min), Deflections Gain (1.9 vs. 1.5 per 60 min), and Rebound (1.5 vs. 1.0 per 60 min), the GK has consistently higher values. This may be due to the GK having more constrained court movement than the GD and not being available to receive a centre pass or feeding the circle.

Finally, an interesting finding for the number of general play turnovers (non-shooting errors) per position, is that the more attacking a player is, i.e., GS, the higher the number is (5.0 per 60 min). This may be because most of their role tends to be with possession of the ball, trying to score or support scoring, and therefore there is a greater opportunity for them to concede more turnovers than defensive areas of the court.

### Case Study: Coach Visualisation Feedback

**Figure 3:**
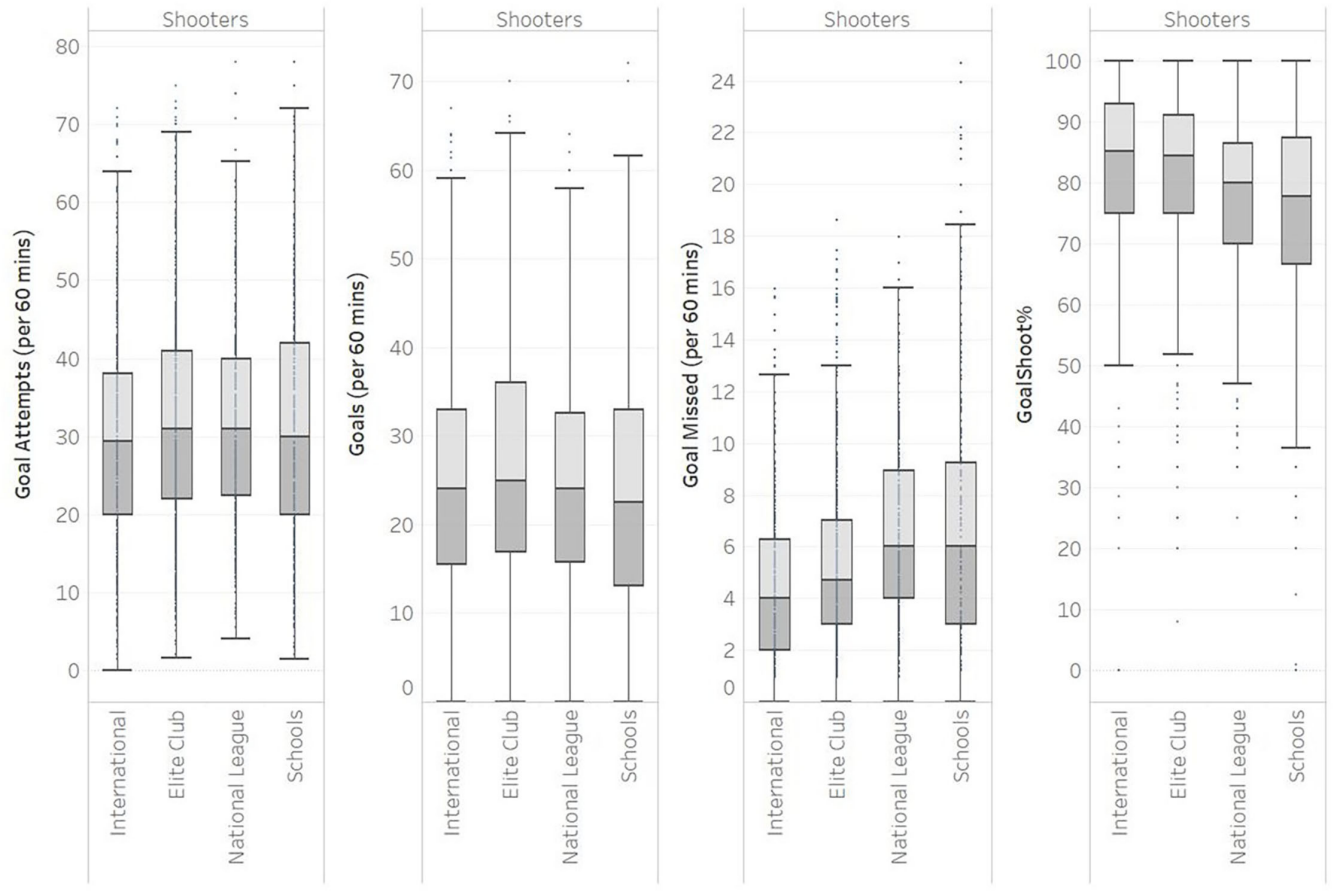
Box and whisker plots of various level of netballs normative data for goal scoring measures.

**Figure 4:**
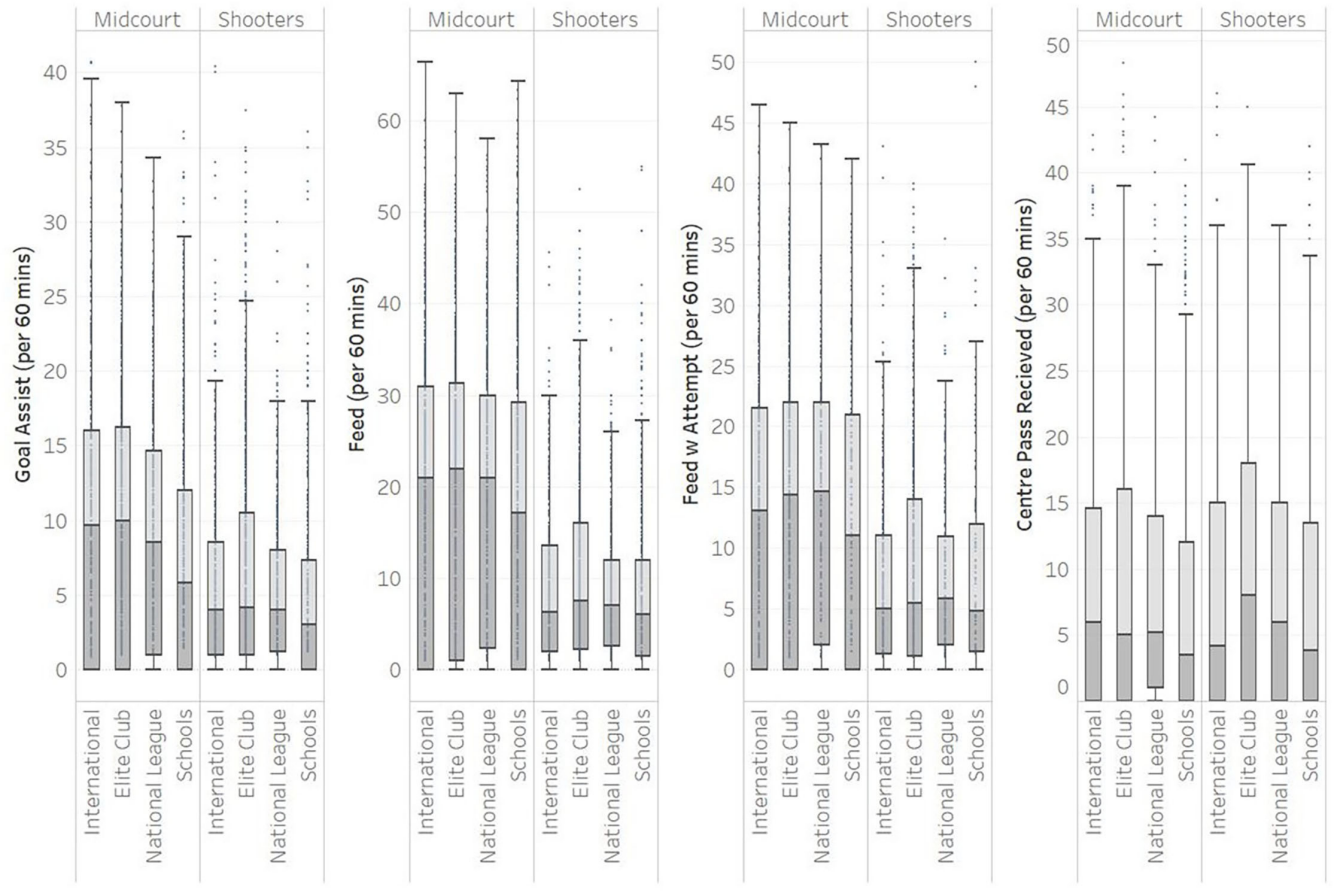
Box and whisker plots of various level of netballs normative data for general play attack measures.

**Figure 5:**
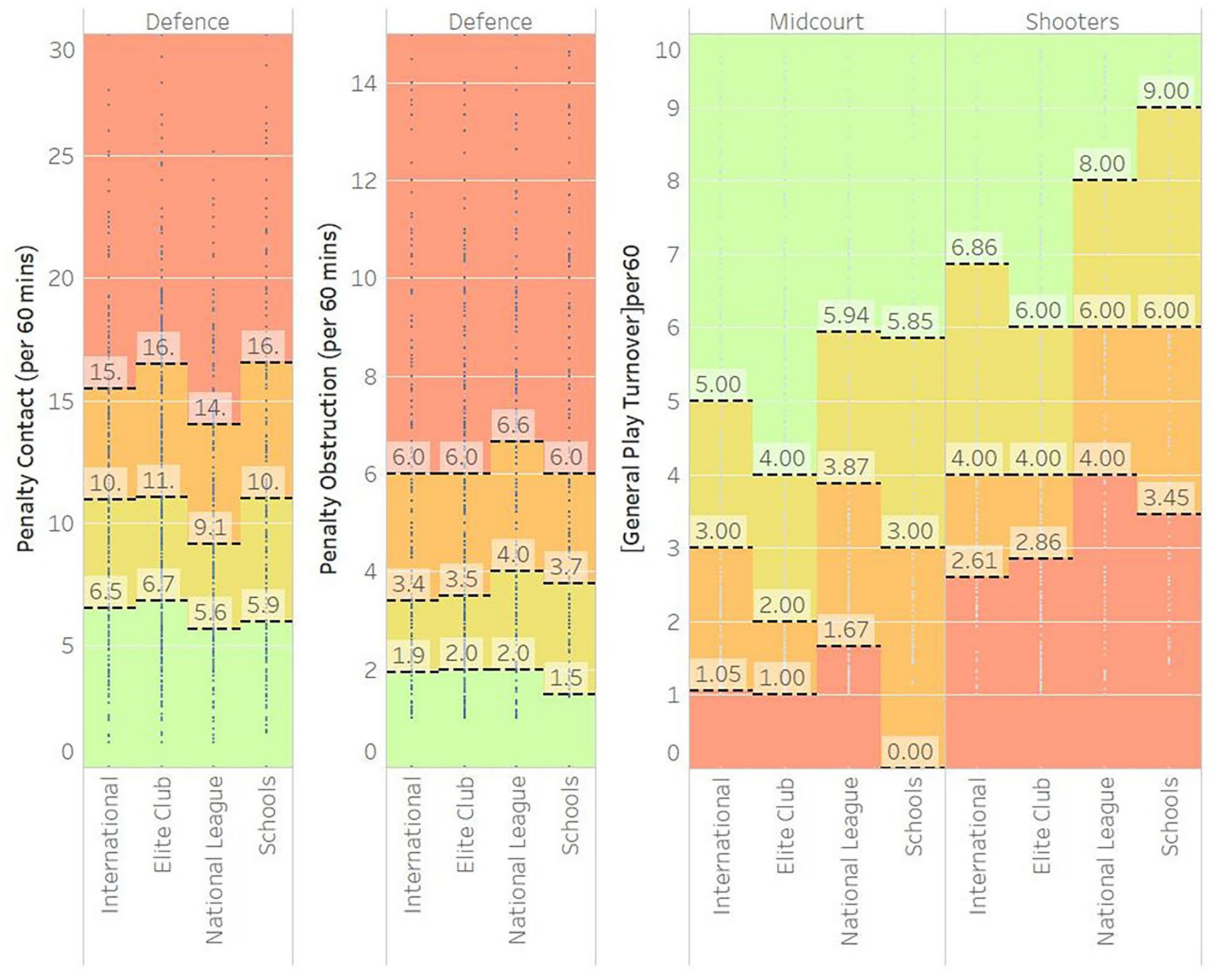
Distribution plots of various level of netballs normative data for general play attack measures.

**Figure 6:**
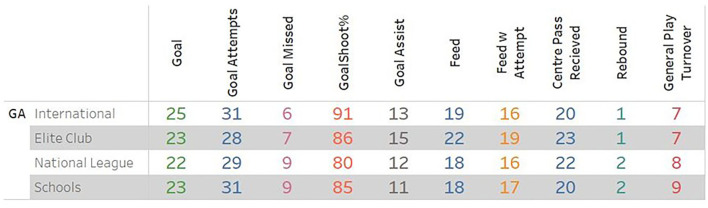
Example of coaches normative table showing 75th percentile values for values for performance indicators.

After consultation with a netball coach, Table 2 and Figures 3–**6** were constructed using filters and categories in the Tableau™ software. These plots and table allow visualisation of the position units, i.e., “shooters,” within different levels of competition. Shooters are the positions GS and GA, “midcourt” are the position WA, C, and WD, while “defenders” are the positions GD and GK. Feedback was given by the coach about how important the need for visualisations that were easy to understand and how easy this would be to explain to the players and other coaches.

**Table 2:** Comparison between the various level of netballs normative data for possession gains measures.

**Possession gains**												
	**Defenders**				**Mid-courters**				**Shooters**			
	**International**	**Elite** **club**	**National** **league**	**Schools**	**International**	**Elite club**	**National league**	**Schools**	**International**	**Elite club**	**National league**	**Schools**
Gains (per 60 mins)												
Percentile (25)	1.9	2.0	2.6	2.1	0.0	0.0	0.0	0.0	0.0	0.0	0.0	0.0
Percentile (50)	3.9	4.0	5.0	4.5	0.0	1.0	1.0	0.0	0.0	0.0	0.0	0.0
Percentile (75)	6.0	6.7	7.7	7.5	2.0	2.0	2.0	2.0	0.0	0.0	0.0	0.0
Intercept (per 60 mins)												
Percentile (25)	0.0	0.0	0.0	0.0	0.0	0.0	0.0	0.0	0.0	0.0	0.0	0.0
Percentile (50)	1.3	1.9	2.0	1.9	0.0	0.0	0.0	0.0	0.0	0.0	0.0	0.0
Percentile (75)	3.0	3.0	3.9	4.4	1.2	1.0	1.9	1.5	0.0	0.0	0.0	0.0
Deflection gain (per 60 mins)												
Percentile (25)	0.0	0.0	0.0	0.0	0.0	0.0	0.0	0.0	0.0	0.0	0.0	0.0
Percentile (50)	1.1	1.1	2.0	2.4	0.0	0.0	0.0	0.0	0.0	0.0	0.0	0.0
Percentile (75)	3.0	3.2	5.0	4.5	1.0	1.0	2.0	2.0	0.0	0.0	0.0	0.0
Deflection no gain (per 60 mins)												
Percentile (25)	1.0	1.9	1.0	0.0	0.0	0.0	0.0	0.0	0.0	0.0	0.0	0.0
Percentile (50)	2.6	3.0	3.0	3.0	0.0	1.0	0.0	0.0	0.0	0.0	0.0	0.0
Percentile (75)	4.0	5.0	4.8	4.5	1.9	2.0	2.0	3.0	0.0	0.0	0.0	0.0
Pick up (per 60 mins)												
Percentile (25)	0.0	0.0	0.0	0.0	0.0	0.0	0.0	0.0	0.0	0.0	0.0	0.0
Percentile (50)	1.0	1.0	1.3	1.5	1.2	1.3	1.4	1.5	0.0	1.0	0.0	0.0
Percentile (75)	2.0	2.0	2.6	2.9	2.6	3.0	3.0	3.0	1.7	2.0	1.5	1.5
Rebound (per 60 mins)												
Percentile (25)	0.0	0.0	0.0	0.0	0.0	0.0	0.0	0.0	0.0	0.0	0.0	0.0
Percentile (50)	1.0	1.0	1.9	1.5	0.0	0.0	0.0	0.0	0.0	1.0	1.4	1.5
Percentile (75)	2.0	2.1	3.2	3.0	0.0	0.0	0.0	0.0	2.0	2.8	3.1	3.0

As presented in Table 2, the defender unit players in international and elite club are consistently lower in the number of “Gains” (3.9 vs. 4.5 per 60 min), “Intercepts” (1.3 vs. 1.9 per 60 min), Deflection Gain (1.1 vs. 2.4 per 60 min), and Pickups (1.0 vs. 1.9 per 60 min) than school and often national league. The coach expressed the desire for a table of this type to be converted into a more visual representation of the data. Box and whisker plots are a visualisation method for looking at the distribution of population data.

Some notable observations from box and whisker plot visualisation (Figure 3) of the normative data include median, or 50th percentile, “goals missed” being lower at international level when compared with school (4 vs. 6 per 60 min), “Goal Shoot%” better at higher levels (85.3 vs. 77.8%).

Box and whisker plots were created for General Play Attack measures with midcourt and shooters separated (Figure 4). This was because their roles for these measures influence the values that might be expected, e.g., Shooters will do less feeding as they also need to be available to receive the feed in-circle. Midcourt players “Centre Pass Receives” were higher in internationals than schools (6 vs. 3.5 per 60 min), and also for “Goal Assists” (9.6 vs. 5.9 per 60 min). Additionally, “Feeds” and “Feeds with Attempt” were consistently higher for the international and elite club level than school. This implies that there are more pre-shot actions at higher levels, yet only a small increase in “Goals” (24 vs. 22.5 per 60 min). This could indicate that there is better execution of these skills at the international and elite club levels, which is reflected in the Defensive players data. The coach felt that these plots were not easy to understand and concept of quartiles was one, they needed more education around.

In Figure 5, the Distribution Plots were used to display error measures with colours, shaded from red for less desirable performance, to green for preferable performance, shading the different 25th, 50th, and 75th percentile boundaries. Although not distinctly measurable, there appears to be an overall visual trend for general play turnovers (GPT) to increase as performance went down the levels, i.e., from international down to schools. It was however clear that the shooters had high GPTs than midcourters.

Finally, the coach communicated that simple table with a benchmark number be produced (Figure 6). The concept of the 75th percentile, or upper quartile, was agreed upon as a benchmark by the coach, as it is represented as an attainable number for players if they improved. This is somewhat subjective, however, aligned with the beliefs of the coach giving a level of ecological validity.

## Discussion

### Workflow and Automation

The purpose of this paper was to develop and explain an automated process for presenting and manipulating normative data for netball matches across various populations. It was proposed that this process should allow filtering of data dimensions (independent variables) and measures (dependent variables). As a result, two data tables (Tables 1, 2) were presented to not only demonstrate the functionality of the process but also to provide insights into some of the characteristics of the population data. The process followed several stages, such as data acquisition, data preparation and cleaning, and upload and consumption.

The data acquisition stage of this process did use some automation *via* the XML file upload, however, to create a large historical dataset, manual data scraping was required. Future data acquisition into this process could, however, be fully automated.

### Normative Data

The results of Table 1 showed percentiles, or quartile ranges, for 22 performance indicators identified by as being highly valid to coach and player decision-making. Table 1 results help describe the characteristics of each position with some being more specialized. Examples of this were GS's and GK players having the highest values for shooting and defensive turnovers, respectively. GA, WA, WD, and GD were represented in a greater number of measures, i.e., Goal Assists, Feeds, Feeds w Attempt, Centre Pass Receives, etc., than other positions, meaning they were involved in more aspects of the game. The C position was the most generalised with representation in most measures except those within the circle. This representation tended to be as a low to moderate frequency for all measures.

With the normative data measures presented a coach could evaluate the performance of their team for strengths and weaknesses by looking at the performances that are either above or below the median (50% percentile). This could assist in decision-making around areas for improvement, or strengths that could be played to.

### Case Study

To demonstrate the flexibility of the tool and to assess its face (Middleton, 2020) (Middleton, 2020)and ecological validity (Ransdell, 1993), (Davids, 1988), the consultation was undertaken with an elite netball coach to determine what groupings of positions, levels of competition, and variables would be useful when applying this tool to coaching. Table 2 was constructed to demonstrate the differences on the normative values for international, elite club, national club, and school levels. Player positions were combined into “units” so that the players could understand the broader requirements of different areas of the court.

With Table 2, a coach or selector could consider player pathways and relative strengths of developing players. An example of this could be that if a GA player has a goal shooting volume similar to that of a GS, then they may be able to change positions and still meet the requirements of the more specialised GS position.

Some differences between the levels were observed in the case study (Table 2). These described the higher levels of competition (international and elite club) as having better goal accuracy, more pre-circle movements, and less turnovers conceded. This could imply that as the players develop and move to the higher levels, they tend to be more accurate with their ball handling skill, play faster with more actions, and have better ball retention.

## Conclusion

The overall objectives of this paper were to not only produce a live tool that could display data to coaches during the matches but also demonstrate this with a normative dataset, that presents useful contextualization to netball coaches and players about their performance statistics. Interesting insights into the normal values for netball player performances were found, with differences between the specialised and more generalised positions observed. Finally, through consultation with expert coaches, a simple visual representation of benchmark statistics was developed. This simple table (Figure 6) was seen to have the best ecological validity for the coach.

## Data Availability Statement

The raw data supporting the conclusions of this article will be made available by the authors, without undue reservation.

## Author Contributions

All authors listed have made a substantial, direct and intellectual contribution to the work, and approved it for publication.

## Funding

AUT SPRINZ have approved half of the fee and Otago Polytechnic have approved to pay the second half of the fee.

## Conflict of Interest

The authors declare that the research was conducted in the absence of any commercial or financial relationships that could be construed as a potential conflict of interest.

## Publisher's Note

All claims expressed in this article are solely those of the authors and do not necessarily represent those of their affiliated organizations, or those of the publisher, the editors and the reviewers. Any product that may be evaluated in this article, or claim that may be made by its manufacturer, is not guaranteed or endorsed by the publisher.
